# Using mobile technology in assessment of entrustable professional activities in undergraduate medical education

**DOI:** 10.1007/s40037-020-00618-9

**Published:** 2020-10-23

**Authors:** Norah Duggan, Vernon R. Curran, Nicholas A. Fairbridge, Diana Deacon, Heidi Coombs, Katherine Stringer, Stephen Pennell

**Affiliations:** Faculty of Medicine, Memorial University, St. John’s, Newfoundland Canada

**Keywords:** Mobile apps, Workplace assessment, Entrustable professional activities, Undergraduate medical education

## Abstract

**Background:**

The adoption of competency-based medical education requires objective assessments of a learner’s capability to carry out clinical tasks within workplace-based learning settings. This study involved an evaluation of the use of mobile technology to record entrustable professional activity assessments in an undergraduate clerkship curriculum.

**Approach:**

A paper-based form was adapted to a mobile platform called eClinic Card. Students documented workplace-based assessments throughout core clerkship and preceptors confirmed accuracy via mobile phones. Assessment scores for the 2017–2018 academic year were collated and analyzed for all core rotations, and preceptors and students were surveyed regarding the mobile assessment experience.

**Evaluation:**

The mobile system enabled 80 students and 624 preceptors to document 6850 assessment submissions across 47 clinical sites over a 48-week core clerkship curriculum. Students’ scores demonstrated progressive improvement across all entrustable professional activities with stage-appropriate levels of independence reported by end of core clerkship. Preceptors and students were satisfied with ease of use and dependability of the mobile assessment platform; however, students felt quality of formative coaching feedback could be improved.

**Reflection:**

Our preliminary evaluation suggests the use of mobile technology to assess entrustable professional activity achievement across a core clerkship curriculum is a feasible and acceptable modality for workplace-based assessment. The use of mobile technology supported a programmatic assessment approach. However, meaningful coaching feedback, as well as faculty development and support, emerged as key factors influencing successful adoption and usage of entrustable professional activities within an undergraduate medical curriculum.

**Electronic supplementary material:**

The online version of this article (10.1007/s40037-020-00618-9) contains supplementary material, which is available to authorized users.

## Background and need for innovation

Direct observation of clinical skills is a key component of medical education. However, there is growing realization that effective use of workplace-based assessments in the medical workplace varies significantly when put into practice. Accreditation standards require documentation that students receive regular, timely, formative feedback [1]; yet preceptor compliance may be challenging, direct observation may occur infrequently, and assessors frequently rely on distant recollections that may not accurately capture clinical performance [2–6].

According to Massie and Ali [7], there is widespread cynicism and negativity towards workplace-based assessments, including a poor understanding of the purpose, insufficient time for assessments, and inadequate training. Students report assessors often have an incomplete understanding of workplace-based assessments, and students themselves may also not fully understand these assessments or the formative intent of such tools [8]. Workplace-based assessments are often viewed as a low priority, and students report a lack of feedback conversations or that feedback was unspecific and/or without justification [8].

Ferenchick et al. [9] suggest flexible assessment tools are needed during routine clinical workflow, and a number of authors propose that mobile technologies offer potential options for more flexible modes of workplace-based assessments [9–11]. Internet-enabled mobile devices such as smartphones and tablets have become ubiquitous, and are being used with increasing frequency in medical education. Torre et al. [12, 13] found mini-clinical evaluation exercise (mini-CEX) assessments hosted on a mobile device to be a feasible method to record direct observations of students’ clinical skills in a timely and efficient manner in both inpatient and outpatient settings. Coulby et al. [14] examined a personal digital assistant-based mini-CEX assessment amongst final-year undergraduate medical students and found an increased level of descriptive feedback, allowing students to improve their skills during the placement.

## Goal of innovation

Entrustable professional activities have been put forward as a means to improve workplace-based assessments by operationalizing competencies and milestones in the clinical setting. Entrustable professional activities have been described as tasks or responsibilities that sufficiently competent learners are expected to perform without direct supervision [15]. We introduced an assessment process across the clerkship phase of our undergraduate Doctor of Medicine (MD) curriculum that involved the assessment of entrustable professional activities [16]. Clinic card forms were developed to assess medical student progression towards entrustable professional activity achievement, and previous work demonstrated the utility and educational benefits of such forms [16]. However, our experiences were that paper-based clinic cards met educational and accreditation objectives, but required significant resources when collating data for programmatic assessment review. We introduced eClinic Cards as a means to enhance the process of workplace-based assessment of the entrustable professional activity achievement of medical students across 47 clinical sites of our 48-week core clerkship program.

## Steps taken for development and implementation of innovation

**Fig. 1:**
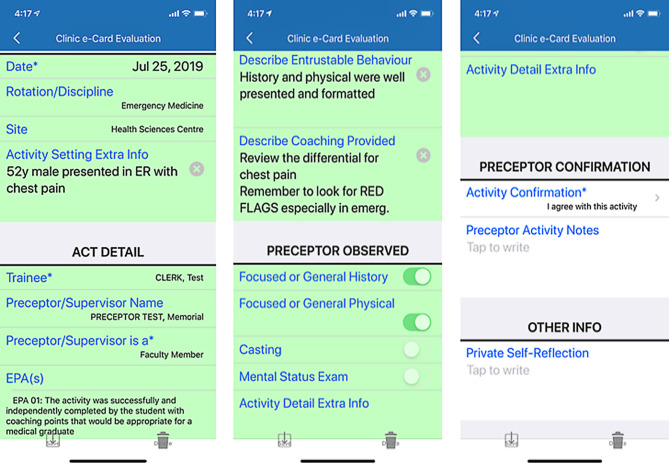
Screen captures of eClinic Card (*T‑Res* *2 Clinic e‑Card*) app

Memorial University offers a 4-year undergraduate MD program. During clerkship, students train in hospital and community settings throughout affiliated teaching sites in Newfoundland and Labrador, New Brunswick, and other jurisdictions. Core rotations include anesthesia, emergency medicine, internal medicine, obstetrics/gynecology, pediatrics, psychiatry, rural family medicine, and surgery. We introduced a programmatic assessment process across clerkship and introduced an eClinic Card app that adapted a paper-based clinic card form [16] into an electronic format (Fig. 1).

The eClinic Card included entrustable professional activity statements identified by the Association of Faculties of Medicine of Canada (activities 1–12) and the Association of American Medical Colleges (activity 13) (Tab. 1, online). The forms also allowed open-ended narrative feedback comments that students were responsible for documenting based on feedback they received during the observation period.

The eClinic Card forms were created using the T‑Res 2 app, a proprietary app found on the iOS App Store and Android Google Play. Both students and preceptors installed the app on their own device. Students were expected to approach their preceptor immediately after an activity to obtain verbal feedback on performance, and then complete the form using their own mobile devices. Once submitted, the system automatically provided a notification and a copy to the preceptor for confirmation. Preceptors had the option to add additional narrative comments at the approval stage. Final assessment data was exportable from the T‑Res platform in Excel file format and converted to PDF file reports for review by clerkship coordinators and to evaluate entrustable professional activity completion.

Medical students undertaking core clerkship (*n* = 80) were required to complete a minimum number of clinic cards per core rotation (Tab. 2, online). A small subset of students (*n* = 10) participated in a longitudinal emergency medicine placement that included an equivalent number of clinic hours. Preceptors included postgraduate medical residents and clinical faculty. Users were oriented to the eClinic Card app and assessment process through formal workshops, academic half-days, grand rounds, one-on-one coaching or through webinars. Online handbooks were produced and disseminated that provided general information on the role of the clinic cards in clerkship assessment and examples of behaviors for each entrustable professional activity.

## Evaluation of innovation

We conducted descriptive analyses of the rating scores from the eClinic Card, including relationships of ratings with time and rotation order, the activity assessed, training site and discipline, and preceptor status (resident or faculty). Factors were assessed through ordinal regression to estimate the odds of returning higher or lower entrustable ratings when *p* < 0.01 (Tab. 3, online). Web-based evaluation surveys were distributed to preceptors across core clerkship rotations, and to all participating clerks. The study received ethics approval from the Health Research Ethics Board, Memorial University.

### eClinic Card usage

A total of 624 preceptors approved 6850 eClinic Card forms. Each student recorded an average of 86 cards (SD = 18.5, range 56–194), averaging 2 clinic cards per week or 5 observed activities. Individual students were assessed by between 23 and 77 preceptors (mean 54.4, SD 10.9) over the course of the 48-week core clerkship, and each preceptor assessed approximately 7 students (mean 7.0, SD 8.5, range 1–57) and approved an average total of 11 cards (SD = 13.2, range 1–113).

### Characterization of the eClinic Card adoption

The most common entrustable professional activity assessed, accounting for 19.5% of all assessments, was activity 1, to obtain a history and perform a physical, and the least assessed at 3.6% was activity 10, quality improvement initiatives. Most students (>80%) completed 5 or more assessments for each of the 13 core entrustable professional activities. Students improved their relative odds of recording higher scores by nearly 50% (1.48 × increased odds) every 2 weeks, and these improvements were cumulative across the 48-week program (Tab. 3, online). Novice clerks were entrusted with independence in 28.5% of initial clinical encounters and with progressive improvement to 97.9% by the end of core clerkship. There were no significant differences in entrustability odds or progression through clerkship when comparing the 13 core entrustable professional activities, and scores were not influenced by whether the preceptor was a resident or clinical faculty (Tab. 3, online).

A comparison of the paper-based [16] and electronic processes was feasible only for the core surgery rotation due to resource limitations in converting paper-based documentation into a de-identified database. While both systems met accreditation standards, we noted a reduction in the number of assessments logged through the mobile platform. Card submissions decreased by 22.4%, and the proportion of cards containing coaching recommendations decreased by 16.2%, resulting in 35% fewer coaching records. The paper cards recorded an average of 6.2 assessed activities per card, while the eClinic Card returned an average of 3.1, a 60.5% reduction in documented activities. The transition to a mobile platform coincided with a 33.3% reduction in the number of participating preceptors in the surgery rotation.

### Student and preceptor satisfaction

Tab. 4 (online) summarizes the results of the survey of students (response rate = 21.5%, *n* = 17/79) and preceptors (response rate = 25.8%, *n* = 81/314). A larger proportion of respondents agreed or strongly agreed that the eClinic Card app was ‘easy to use’ (students = 47.1%, preceptors = 64.2%) and ‘dependable to use’ (students = 47.1%, preceptors = 80.3%), and a majority of preceptor respondents reported the eClinic Card app was ‘easy to review and confirm information’ (66.7%). However, a larger proportion of student respondents disagreed or strongly disagreed that ‘the clinic card process helped to facilitate appropriate coaching feedback’ (88.2%), ‘helped to identify areas of strength and areas for further growth’ (58.8%), or ‘coaching feedback and narrative comments were helpful to learning’ (52.9%). As well, a larger proportion of preceptor respondents were neutral, disagreed or strongly disagreed that ‘the clinic card process helped to facilitate the conversation and coaching feedback’ (50.6%). A majority of student respondents (88.2%) also disagreed that preceptors responded to cards in a timely fashion. An analysis of ‘time to submission’ revealed that student submissions were often delayed for days after the assessed interaction (mean = 4.9 days, SD = 9.7; median = 1.2 days), and preceptor review and approval of cards was regularly delayed by weeks to months as well (mean = 13.2 days, SD = 27.8; median = 3.0 days).

## Critical reflection

Torre et al. [12, 13] suggest that mobile technology enables timely and efficient approaches for formative and longitudinal workplace-based assessment, while other studies demonstrate that mobile devices make the logistics of assessments easier and have a positive impact on assessors’ ability to provide feedback [9, 10]. In the study of Coulby et al. [14], students found use of mobile technology acted as an ice breaker, encouraged engagement, and facilitated an opportunity for staff and students to initiate a dialogue on their performance.

In our evaluation, the eClinic Card form enabled students to meet the assessment expectations for each core rotation by completing a sufficient level of workplace-based assessments. A greater proportion of observations were recorded for activities focused on collecting information and formulating plans, reflecting findings similar to those of Torre et al. [13], who found the most common focus of encounters was data gathering alone (23%), followed by data gathering/diagnosis/therapy (19%).

Our analysis also indicates there was a significant improvement in students’ workplace-based assessment scores as they progressed in their core clerkship rotations. Similarly, Wagner et al. [17] also found positive linear improvements in performance scores with the use of mobile end-of-rotation performance evaluations. Our analysis did indicate a fair delay in completing mobile workplace-based assessments, where only 1 in 6 interactions resulted in the completion of an eClinic Card within 24 h of the encounter. Yet, learners report that immediate and detailed feedback is the most valuable feature of workplace-based assessments [7]. Our students did not find the narrative feedback to be helpful in their learning, and this coincided with a large proportion of cards (41%) lacking coaching comments identifying specific, detailed or actionable areas for improvement. In a recent study, Young et al. [21] observed that when faculty directly submitted comments using a mobile app to capture data on entrustable professional activities, feedback was received in a timely and frequent manner, and residents appreciated receiving the feedback within minutes of the encounter. It is possible that the change from preceptor-led comments with paper-based cards to student-led summarization of preceptor comments with the eClinic Card may have influenced timely submissions, extent, and quality of the written feedback.

Several authors have reported the importance of functionality and ease of use with the introduction of mobile workplace-based assessment systems [12, 13, 18, 19], and recent work [21] has also confirmed ease of use and intuitiveness of a mobile app to capture data on entrustable professional activities in postgraduate workplace-based assessment [20]. In our study, students were satisfied with the use of the mobile app and found it dependable and easy to use. Preceptors reported that the eClinic Card made it easier to review and confirm assessments for students, and the collation and reporting of scores was more efficient.

A majority of preceptors felt that further faculty development would have been helpful in preparing them for the use of the eClinic Card, their approach to coaching, and providing feedback to students. Cendan et al. [18] also found that faculty development was important in introducing mobile workplace-based assessment, and Norman et al. [19] found preceptors were unfamiliar with the use of personal digital assistants for workplace-based assessment, and many lacked confidence in the technology. According to Sandars and Dearnley [20], training is essential for all users when introducing mobile work-based assessment and should be provided in a variety of formats (face-to-face and online).

Our experiences indicate that use of mobile technology and an electronic submission process for assessing undergraduate entrustable professional activities achievement is feasible. The mobile eClinic Card app was found easy to use, and there was general acceptance of the use of mobile technology for workplace-based assessments. The use of a mobile system also enhanced the efficiency by which assessment scores could be summarized and reported in support of a programmatic assessment approach. However, the process of fostering greater interaction and more immediate feedback to students from preceptors—an intention for adopting the mobile eClinic Card app—did not appear to materialize. The process of preceptor-led versus student-led feedback submission as enabling or inhibiting meaningful and timely feedback provision when using mobile apps does warrant further investigation.

## Caption Electronic Supplementary Material

Tab. 1: Entrustable professional activities in core clerkship assessment, 2017–2018

Tab. 2: Clinic card characteristics by rotation

Tab. 3: Proportional odds of scoring in the entrustable professional activity

Tab. 4: Student and preceptor satisfaction with the eClinic Card Entrustable professional activities in core clerkship assessment, 2017–201
