# Supplementary material for: Using mobile technology in assessment of entrustable professional activities in undergraduate medical education
Source: Perspect Med Educ. 2020 Oct 23;10(6):373–7. doi: 10.1007/s40037-020-00618-9 (PMC8633342; doi:10.1007/s40037-020-00618-9)
Supplement: Supplementary file 1 — Tab. 1: Entrustable professional activities in core clerkship assessment, 2017–2018 [file 40037_2020_618_MOESM1_ESM.docx]

**Table 1.** Entrustable professional activities in core clerkship assessment, 2017-2018

| **Number** | **Activity descriptor** |
| --- | --- |
| 1 | Obtain a history and perform a physical examination adapted to the patient’s clinical situation. |
| 2 | Formulate and justify a prioritized differential diagnosis. |
| 3 | Formulate an initial plan of investigation based on the diagnostic hypotheses. |
| 4 | Interpret and communicate results of common diagnostic and screening tests. |
| 5 | Formulate, communicate, and implement management plans. |
| 6 | Present oral and written reports that document a clinical encounter. |
| 7 | Provide and receive the handover in transitions of care. |
| 8 | Recognize a patient requiring urgent or emergent care, provide initial management, and seek help. |
| 9 | Communicate in difficult situations. |
| 10 | Participate in health quality improvement initiatives. |
| 11 | Perform general procedures of a physician. |
| 12 | Educate patients on disease management, health promotion, and preventative medicine. |
| 13 | Collaborate as a member of an interprofessional team. |
