# Supplementary material for: Using mobile technology in assessment of entrustable professional activities in undergraduate medical education
Source: Perspect Med Educ. 2020 Oct 23;10(6):373–7. doi: 10.1007/s40037-020-00618-9 (PMC8633342; doi:10.1007/s40037-020-00618-9)
Supplement: Supplementary file 2 — Tab. 2: Clinic card characteristics by rotation [file 40037_2020_618_MOESM2_ESM.docx]

**Table 2** Clinic card characteristics by rotation

|  | | **Rotation length**  **(weeks)** | **Cards required** | **Average cards completed** | **Preceptors** | **% Faculty** | **% Resident** |
| --- | --- | --- | --- | --- | --- | --- | --- |
|  | Emergency medicine | 2 | 9 | 9.3 | 92 | 97.1 | 2.9 |
|  | Obstetrics/gynecology | 6 | 17 | 18.5 | 94 | 54.8 | 45.2 |
|  | Internal medicine | 10 | 20 | 19.2 | 231 | 21.8 | 78.2 |
|  | Pediatrics | 8 | 18 | 16.0 | 98 | 89.9 | 10.1 |
|  | Psychiatry | 6 | 12 | 8.3 | 57 | 60.0 | 40.0 |
|  | Rural family medicine | 8 | 8 | 8.0 | 70 | 94.4 | 5.6 |
|  | Surgery | 8 | 7 | 6.4 | 88 | 53.9 | 46.1 |
| Total | | 48 | 91 | 85.7 | 624^a^ | 62.7 | 37.3 |

^a^A subset of preceptors worked within multiple disciplines and assessed clerks from two or more discipline rotations.
