# Supplementary material for: Using mobile technology in assessment of entrustable professional activities in undergraduate medical education
Source: Perspect Med Educ. 2020 Oct 23;10(6):373–7. doi: 10.1007/s40037-020-00618-9 (PMC8633342; doi:10.1007/s40037-020-00618-9)
Supplement: Supplementary file 3 — Tab. 3: Proportional odds of scoring in the entrustable professional activity [file 40037_2020_618_MOESM3_ESM.docx]

**Table 3** Proportional odds of scoring in the entrustable professional activity (*EPA*) assessments

|  | **Estimate** | **Std. error** | **Wald** | ***df*** | **Sig.** | **Relative odds** | **Lower bound** | **Upper bound** |
| --- | --- | --- | --- | --- | --- | --- | --- | --- |
| Time (biweekly) | 0.392 | 0.016 | 576.441 | 1 | 0.000 | 1.481 | 1.434 | 1.529 |
| Rotation order | -0.912 | 0.066 | 189.108 | 1 | 0.000 | 0.402 | 0.353 | 0.457 |
| Emergency medicine > surgery | 1.651 | 0.579 | 8.126 | 1 | 0.004 | 5.213 | 1.675 | 16.224 |
| Internal medicine > surgery | 1.452 | 0.380 | 14.629 | 1 | 0.000 | 4.273 | 2.030 | 8.993 |
| Other disciplines ~ surgery | Not significant | | | | |  | | |
| EPA type | Not significant | | | | |  | | |
| Longitudinal clerkship | Not significant | | | | |  | | |
| Preceptor status | Not significant | | | | |  | | |
| Students |  |  |  |  |  | Personal scores not disclosed | | |
| Preceptors |  |  |  |  |  | Personal scores not disclosed | | |
| Clinical sites |  |  |  |  |  | Clinic scores not disclosed | | |
| Overall model fit was appropriate [chi-squared (742) = 7564.8, *p*<0.000], as was the goodness-of-fit [Pearson (33,790) = 33,101.8, *p*=0.996; deviance (33,790) = 14,240.2, *p*=1.00]. The model pseudo-*R*^2^ was between 0.338 (Cox and Snell) and 0.478 (Nagelkerke). Significance (*Sig.*) was considered where *p*<0.01 The proportional odds of EPA scoring for individual students, preceptors, and each clinical site were included in the ordinal regression calculations but not disclosed here | | | | | | | | |
