# Supplementary material for: Using mobile technology in assessment of entrustable professional activities in undergraduate medical education
Source: Perspect Med Educ. 2020 Oct 23;10(6):373–7. doi: 10.1007/s40037-020-00618-9 (PMC8633342; doi:10.1007/s40037-020-00618-9)
Supplement: Supplementary file 4 — Tab. 4: Student and preceptor satisfaction with the eClinic Card Entrustable professional activities in core clerkship assessment, 2017–201 [file 40037_2020_618_MOESM4_ESM.docx]

**Table 4** Student and preceptor satisfaction with the eClinic Card *(T-Res 2 Clinic e-Card)*

| **Student satisfaction** | | | | | |
| --- | --- | --- | --- | --- | --- |
| **Item** | **Strongly disagree** | **Disagree** | **Neither agree nor disagree** | **Agree** | **Strongly agree** |
| I found it easy to access the T-Res 2 Clinic e-Card app. | 5.9% | 5.9% | 17.7% | 58.8% | 11.8% |
| I found it easy to use the T-Res 2 Clinic e-Card app. | 11.8% | 11.8% | 29.4% | 41.2% | 5.9% |
| The T-Res 2 Clinic e-Card app was dependable to use (e.g., limited technical issues). | 17.7% | 17.7% | 17.7% | 47.1% | 0.0% |
| I found it easy to listen and report the conversation with the preceptor accurately. | 52.9% | 23.5% | 11.8% | 11.8% | 0.0% |
| The clinic card process helped to facilitate appropriate coaching feedback between the preceptor and me. | 47.1% | 41.2% | 5.9% | 5.8% | 0.0% |
| The preceptors responded to the clinic card in a timely fashion. | 58.8% | 29.4% | 0.0% | 11.8% | 0.0% |
| The clinic card coaching process helped me identify areas of strength and areas for further growth in my clinical practice. | 23.5% | 35.3% | 23.5% | 17.7% | 0.0% |
| The coaching feedback and narrative comments I recorded were helpful to my learning. | 11.8% | 41.2% | 35.3% | 11.8% | 0.0% |
| Overall, the clinic card coaching process contributed to a positive phase 4 learning experience. | 41.2% | 11.8% | 41.2% | 5.9% | 0.0% |
| *n*=17 | | | | | |

| **Preceptor satisfaction** |
| --- |
| \| **Item** \| **Strongly disagree** \| **Disagree** \| **Neither agree nor disagree** \| **Agree** \| **Strongly agree** \| \| --- \| --- \| --- \| --- \| --- \| --- \| \| There was adequate faculty development for the implementation and use of entrustable professional activities in assessment in phase 4 clerkship. \| 7.4% \| 23.5% \| 45.7% \| 18.5% \| 4.9% \| \| There was adequate faculty development for the use of the clinic card process. \| 7.4% \| 25.9% \| 43.2% \| 18.5% \| 4.9% \| \| I found it easy to use the T-Res 2 Clinic e-Card app/website. \| 2.5% \| 11.1% \| 22.2% \| 44.4% \| 19.8% \| \| The T-Res 2 Clinic e-Card app/website was dependable to use (e.g., limited technical issues). \| 1.2% \| 7.4% \| 11.1% \| 61.7% \| 18.5% \| \| The clinic card process helped to facilitate the conversation and coaching feedback between the student and myself. \| 6.2% \| 18.5% \| 25.9% \| 38.3% \| 11.1% \| \| I received notification of clinic e-cards to review in a timely fashion. \| 2.5% \| 4.9% \| 13.6% \| 54.3% \| 24.7% \| \| I found it easy to review and confirm the information as recorded by the student. \| 3.7% \| 11.1% \| 18.5% \| 44.4% \| 22.2% \| \| I found it easy to add notes to the e-card before returning it to the student to modify. \| 5.0% \| 12.5% \| 20.0% \| 45.0% \| 17.5% \| \| Overall, the clinic card coaching process contributed to a positive phase 4 preceptor experience. \| 4.9% \| 13.6% \| 35.8% \| 34.6% \| 11.1% \| \| *n*=81 \| \| \| \| \| \| |
